# Extracellular Vesicles as Biomarkers and Therapeutic Tools: From Pre-Clinical to Clinical Applications

**DOI:** 10.3390/biology10050359

**Published:** 2021-04-23

**Authors:** Maria Chiara Ciferri, Rodolfo Quarto, Roberta Tasso

**Affiliations:** 1 Department of Experimental Medicine, University of Genova, 16132 Genova, Italy; S4971023@studenti.unige.it; 2 U.O. Cellular Oncology, IRCCS Ospedale Policlinico San Martino, 16132 Genova, Italy

**Keywords:** extracellular vesicles, exosomes, cancer, biomarkers, liquid biopsy, therapy, clinical trials

## Abstract

**Simple Summary:**

Extracellular vesicles (EVs) are membrane-bound vesicles released by all cell types, differing in biogenesis, physical characteristics, and contents. Due to their central role in intercellular communication and their variable cargo, EVs are involved in several biological processes. The possibility of isolating them from different biofluids makes EVs valuable biomarkers to be analyzed for the diagnosis or prognosis of several conditions. Moreover, these natural nanoparticles have been investigated as therapeutic tools in many pathological conditions. In this context, EVs have shown innate immunosuppressive and anti-inflammatory properties when isolated from stem/progenitor cells and have also been considered vehicles to be edited for drug delivery. The aim of the review is to report some of the pre-clinical and clinical studies distinguishing those in which EVs have been examined as biomarkers from those in which they have been used as therapeutics.

**Abstract:**

Extracellular vesicles (EVs) are ubiquitous masters of intercellular communication, being detectable in tissues, circulation, and body fluids. Their complex cargo reflects the (patho)physiologic status of the cells from which they originate. Due to these properties, the potential of EVs, and in particular exosomes, to serve as biomarkers or therapeutics has grown exponentially over the past decade. On one side, numerous studies have demonstrated that EV-associated nucleic acids and proteins are implicated in cancer progression, as well as neurodegenerative, infectious, and autoimmune disorders. On the other, the therapeutic use of EVs secreted by various cell types, and in particular stem/progenitor cells, present significant advantages in comparison to the corresponding parental cells, such as the less complex production and storage conditions. In this review, we examine some of the major pre-clinical studies dealing with EVs and exosomes, that led to the development of numerous completed clinical trials.

## 1. Introduction

Extracellular vesicles (EVs) are a heterogeneous group of membrane-bound vesicles released by all cell types and containing cell-derived biomolecules [1]. Increasing evidence indicates that the biogenesis, the physical characteristics, as well as the different cargos are at the basis of EV heterogeneity [2]. EVs can be roughly divided into two main categories: exosomes and microvesicles [3]. The term exosome refers to intraluminal vesicles with a diameter ranging from 30 to 100 nm, formed during the maturation of multivesicular endosomes (MVE), and released when MVE fuse with the cell plasma membrane. Microvesicles (MVs) have been recently reported to play a role in intercellular communication among various cell types. Their size ranges from 50 to 1000 nm or more. MV biogenesis is completely different from that of exosomes. They are generated by the budding of the plasma membrane and are directly released in the extracellular space [4].

Nowadays, the effects specifically exerted by one EV subpopulation over another are still unclear. This is in part due to their overlapping size and variable cargos, which do not allow to precisely discriminate between them. In 2016, Kowal and colleagues suggested to avoid the use of either the term exosome or microvesicle in the absence of a clear demonstration of their intracellular origin [5]. Following, the ISEV (International Society For Extracellular Vesicles) board members published a position paper stating that the use of operational terms for EV subtypes that refer to: (i) physical characteristics (size or density), (ii) biochemical composition, or (iii) description of conditions or cell of origin, is strongly encouraged in the place of the terms exosome and microvesicle [6].

**Figure 1:**
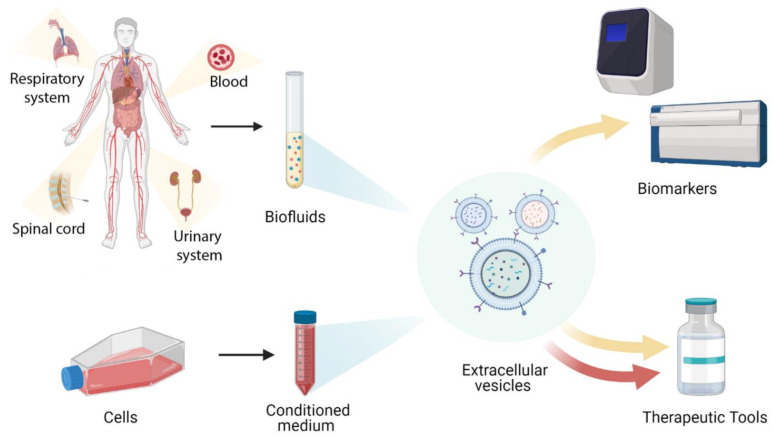
Schematics depicting the possible use of Extracellular Vesicles as biomarkers or therapeutics in both pre-clinic and clinic applications.

Given their role as vehicles of intercellular communication, EVs are involved in many (patho)physiological processes [7]. This characteristic, together with the molecular composition that reflects the status of the producing cell, make EVs exploitable tools for diagnostic and therapeutic purposes [8,9] (Figure 1). This review aims at summarizing the most recent literature on EVs as disease biomarkers, as well as candidates for cell-free therapeutic approaches. In the first part of the review, we will focus on the more recent pre-clinical studies. Particular attention will be then given to clinical trial data in the last part of the review.

### 1.1. EVs as Prognostic or Diagnostic Tools in Pre-Clinical Studies

In the last 20 years, circulating EVs, and in particular exosomes, have been extensively investigated as biomarkers in liquid biopsy [10]. The interest of the scientific community on this topic has grown exponentially mainly because EVs are highly stable and can be found in all body fluids [11]. However, despite the progresses made in EV isolation and characterization and the guidelines recently published [6], in some of the articles cited in this review, a clear definition of the EV subtypes analyzed is lacking. The main biological fluids in which circulating EVs have been identified are blood (plasma or serum), urine, saliva, breast milk, amniotic fluid, cerebrospinal fluid, pleural effusion, and bronchoalveolar lavage fluid [12].

Noninvasive blood biopsy has gained significant interest mainly in the context of tumor diagnosis and treatment response assessment [13]. Tumor-derived EVs are related to tumor progression, metastasis, and immune evasion [14]. One of the earliest evidences of EVs as source of tumor-rejection antigens dates back to 2004 [15]. Patients with peritoneal or pleural carcinomatosis associated with ascites or pleural effusions were included in the study, and heterogeneous vesicles exposing tumor antigens were identified in these malignant effusions. Since that date, thousands of experimental data have accumulated and EVs have been investigated in almost all aspects of cancer. For instance, it has been reported that glypican-1 (GPC1), a cell surface proteoglycan, is specifically expressed by exosomes isolated from the serum of patients with pancreas cancer, and levels of GPC1+ exosomes correlates with tumor burden and survival in patients pre- and post-surgical tumor resection [16]. Apart from primary tumors, EVs have been also investigated for the early detection of metastasis. Two proteins, MDA-9 and GRP78, present a significantly higher expression in the exosomes derived from serum samples of metastatic melanoma patients than those of patients without metastases, indicating that these vesicle-associated proteins can be considered as biomarkers for early detection of metastasis [17]. Moreover, the development of EVs as biomarkers to improve inclusion criteria for the screening of non-small cell lung cancer (NSCLC) patients has been explored, since it could be particularly useful to better assess the risk of intermediate nodules and to refine prognosis [18]. miRNA and long-non coding RNA isolated from the circulating exosomes of NSCLC patients have been demonstrated to mirror the signatures identified in primary tumors [19]. Likewise, the serum level of exosome-associated miR-373 seems to distinguish different breast cancer subtypes, being higher in triple negative than in luminal carcinoma patients [20]. Similar observations have been made considering other types of tumors, such as colorectal cancer [21], nasopharyngeal carcinoma [22], or ovarian cancer [23].

Urinary EV biomarkers have been identified for a variety of kidney and genitourinary tract conditions [24]. In 2013, Alvaro S. and colleagues reported that neutrophil gelatinase-associated lipocalin (NGAL) in urinary exosomes is a better predictor of kidney dysfunction after kidney transplantation than other urinary fractions [25]. More recently, it has been demonstrated that the ratio between the two urinary exosomal proteins polycystin-1 (PC1) and transmembrane protein 2 (TMEM2) could be used to distinguish individuals with polycystic kidney disease-1 (PKD1) mutations from healthy controls [26]. The urinary exosomal WT-1 (Wilms’ tumor-1) has been indicated as a promising noninvasive biomarker with podocyte specificity that can detect early progression and treatment-induced regression of podocyte injury in focal segmental glomerulosclerosis (FSGS) or steroid-sensitive nephrotic syndrome (SSNS) patients [27].

Saliva is considered a valuable diagnostic tool for local and systemic diseases and conditions [28]. As happens in many other pathological conditions, exosomes present in the saliva of patients suffering from oral cancers have different structural characteristics compared to those found in healthy subjects [29]. Byun J. and colleagues identified a specific miRNA, miR-4484, significantly upregulated in the salivary exosomes of patients with oral lichen planus, a chronic inflammatory oral mucosa disease [30]. Moreover, saliva-derived vesicles have been proposed as biomarkers for pancreatic cancer [31], pancreatobiliary tract cancer [32], and lung cancer [33].

de la Torre Gomez C. and colleagues recently published an exhaustive review focusing on EVs derived from human breast milk, stating that they could provide a more specific window into breast milk quality from both an immunological and nutritional point of view [34].

A unique proteomic profile seems to distinguish amniotic fluid–derived exosomes isolated from patients who had spontaneous preterm birth or premature rupture of membranes compared with those who delivered at term [35].

Exosomes from cerebrospinal fluid (CSF) of patients with traumatic brain injury and spinal cord injury have been also investigated as potential biomarkers. NLRP-1- (nucleotide-binding and oligomerization domain (NOD)-like receptor protein-1) and IL-1β- carrying exosomes are described to amplify the inflammatory reaction [36]. In any case, CSF-derived exosomes have been mainly investigated in the context of neurodegenerative diseases, first and foremost Alzheimer’s and Parkinson’s disease. In the postmortem studies of Alzheimer’s disease, CSF-exosomes contained significantly increased levels of total tau and p-tau proteins when compared to the controls [37]. Moreover, two Parkinson’s disease-related proteins, LRRK2 and DJ-1, have been identified in urinary and CSF exosomes [38].

Despite the open challenges associated to the use of EVs, that are mostly related to the difficulties in standardizing the isolation and characterization procedures, this quick roundup of pre-clinical articles indicates that EVs could represent a promising tool for early diagnosis and prognosis of many (patho)physiological conditions.

### 1.2. EVs as Therapeutic Tools in Pre-Clinical Studies

EVs have been extensively investigated as therapeutics in many pathological conditions. In this context, it is particularly important to deeply consider the parental cell characteristics, since they will be, at least in part, transferred to the released vesicles [39]. Due to their intrinsic features, stem/progenitor cells represent the main source of EVs for therapeutic applications. In particular, mesenchymal stromal cells (MSCs) isolated from various tissue sources were extensively explored as EV-donors in many pre-clinical studies, exploiting their strong immunosuppressive and anti-inflammatory properties [40]. One of the first articles dealing with this specific topic has been published in 2010 by Lai R.C. and colleagues [41]. In this paper, MSC-secreted 50- to 100-nm particles were able to reduce infarct size in a mouse model of myocardial ischemia/reperfusion injury. Afterwards, many other studies reported the beneficial effects of MSC-EVs in the context of myocardial infarction, promoting angiogenesis [42], increasing levels of ATP and NADH and decreasing oxidative stress [43]. The therapeutic effects of EVs, and in particular exosomes, derived also from other stem/progenitor cell types in the context of myocardial infarction have been investigated [44,45,46].

A recently published comprehensive meta-analysis of 31 studies summarized the effects of MSC-EVs in improving renal function and reducing cell apoptosis in pre-clinical rodent acute kidney injury (AKI) models [47].

Despite the beneficial outcomes of MSCs in a variety of chronic liver disease models [48], just few articles were published evaluating the role of their corresponding EVs. MSC-EVs encapsulated in PEG-hydrogels exerted anti-fibrotic, anti-inflammatory and pro-regenerative effects in a rat model of chronic liver fibrosis [49]. Another interesting study indicated that human embryonic stem cell-derived MSC-EVs demonstrated immunomodulatory activities comparable to parental cells, and ameliorated cirrhosis in a thioacetamide-induced chronic rat liver injury [50].

MSC-EVs have been also explored as therapeutic agents in many pre-clinical models of acute lung injury (ALI), such as endotoxin-induced ALI [51,52,53], bacteria-induced ALI [54], and ischemia/reperfusion-induced ALI [55], as well as in a model of bronchopulmonary dysplasia [56], and hypoxic pulmonary hypertension [57]. In the latter study, EVs were able to blunt the up-regulation of genes related to inflammation, adaptive immune responses, IFN-γ–mediated signaling, granulocyte and cytokine production.

Finally, MSC-derived EVs were investigated in regenerative medicine settings, such as skin wound healing, osteoarthritis counteracting, and fracture repair. Even in these scenarios, the beneficial outcomes were related to the dampening of the immune responses, and in particular innate immunity, and in the restoration of cytoprotective pathways. Adipose tissue MSC-EVs accelerated the migration and proliferation of keratinocytes and fibroblasts, activating the AKT pathway, and promoting wound healing in an excisional wound-splinting model [58].

Osteoarthritis (OA) is a degenerative joint disorder, that results in persistent pain and disability and high costs to society [59]. It has been demonstrated that MSC-derived exosomes protected cartilage and bone from OA degradation by increasing the expression of the chondrocyte markers type II collagen and aggrecan, reducing catabolic markers such as MMP-13 and ADAMTS5, decreasing inflammatory markers (iNOS), protecting chondrocytes from apoptosis, and blocking macrophage activation [60]. In addition, Zhang S. and co-authors explored the contribution of MSC-exosomes in a rat model of temporomandibular joint OA [61]. Further, in this model, exosomes acted to inhibit pain and degeneration, decreasing inflammation, and enhancing the architecture of subchondral bone. Recently, Tanja Niedermair and co-authors published an interesting paper dealing with the intercellular effects of EVs derived from pathologically altered cells, that could counteract the beneficial effects exerted by healthy MSCs [62]. The authors isolated EVs from osteoblasts of patients with hip OA(coxarthrosis/CA), osteoporosis (OP), or a combination of both (CA/OP) and demonstrated that the viability and differentiation potential of bone marrow-MSCs stimulated in vitro with EVs released by osteoblasts from CA, OP, and CA/OP patients was significantly reduced compared to control groups, suggesting that pathological EVs exerted catabolic effects that should be taken into consideration when harnessing them as therapeutic drugs [62]. EVs secreted by osteocytes have been isolated from the blood, indicating that circulating EVs could represent interesting biomarkers to be investigated, being able to transfer their cargo to distant recipient cells and regulating several biological responses in musculoskeletal disorders [63,64].

Starting from an article published in 2016 by Furuta T. and colleagues [65], an EV-based therapeutic application has been investigated also in the context of fracture repair. In this paper, exosomes isolated from MSC-conditioned medium (CM) were applied in a femur fracture model of CD9−/− mice, a strain that is known to produce reduced levels of exosomes and characterized by a significantly lower bone union rate compared to wild-type mice. EVs were able to rescue the fracture healing retardation in this transgenic mice, and to increase the expression level of bone repair-related cytokines [65]. More recently, MSC-EVs associated with specific scaffolds have been used in rat critical-sized cranial defect models, where they were able to stimulate bone formation and vasculogenesis compared to experimental controls [66,67].

Given the multitude of preclinical studies exploring the efficacy of EVs as biomarkers and therapeutics, specific clinical trials were rapidly launched. An overview of the main vesicle-based clinical trials will be reviewed in the following paragraphs.

## 2. EV-Based Clinical Applications

The database www.ClinicalTrials.gov, accessed on 16 March 2021, has been examined to evaluate the main EV-clinical applications. Currently, 79 trials are registered within the study object “extracellular vesicles” and 208 within “exosomes”. Focusing on those with a “Recruitment Status” belonging to the following categories: “Recruiting”, “Enrolling by invitation”, “Active, not recruiting” and “Completed”, the number of trials decreased to 55 for “extracellular vesicles” and 147 for “exosomes”. Of these, 45 “extracellular vesicles”-associated studies are related to the use of EVs as biomarkers and 10 as therapeutic tools. One hundred and twenty-eight studies are related to the use of “exosomes” as biomarkers and 19 as therapeutics.

The category “biomarkers” includes studies in which vesicles are the primary and most important outcome (“Primary outcome”), as well studies in which vesicles are secondary indicators examined in association with other biomarkers (“Secondary outcome”). The category “therapeutics” comprises trials in which vesicles are both the primary tools and potential targets to be inhibited.

In this review, we will focus only on some of the “completed” clinical studies in which EVs/exosomes are considered as a “primary outcome” (Table 1 and Table 2).

### 2.1. Extracellular Vesicles as Biomarkers in Clinical Applications

EVs have been isolated from several different types of body fluids including plasma, serum, blood, saliva, amniotic fluid, and bronchoalveolar fluid. The isolation and analysis from these biofluids can provide an important support to disease diagnosis and prognosis, as EVs contain information related to the cells of origin [70].

The Catholic University of Lille completed an interventional clinical trial to evaluate if cigarette smoking could alter the miRNA profiles of EVs present in human Bronchoalveolar Lavages (BAL), affecting the physiologic status of neighboring bronchial epithelial cells. The aim of the study was to quantify target mRNA expression in human bronchial epithelial cells exposed to EVs isolated from BAL of 20 smoker and not smoker participants [83].

The English Institute of Cancer Research employed an interventional EV-based liquid biopsy (serum) approach to detect tumor-associated hypoxia (prognostic value). The final goal was to identify patient subpopulations in which the effectiveness of both radiotherapy and chemotherapy was compromised because of the tumor resistance to ionizing radiation. Sixteen participants with proven cancer and four healthy volunteers have been included in this study and the Pimonidazole drug, which has the property of binding exclusively oxygen-starved tissues, has been used to detect the hypoxia condition [71].

EVs have been also considered in the diagnosis of Parkinson Disease (PD) in the “Fox BioNet Project”. Michael J. Fox Foundation for Parkinson’s Research sponsored an observational study with the aim of optimizing an isolation protocol for cerebrospinal fluid (CSF)-derived EVs, to enrich and increase the detection of specific PD-associated mutations in *LRRK2* (Leucine-rich repeat kinase 2). A combination of 36 people have been enrolled in this study, combining PD patients and healthy controls in non-specified proportions [73].

Another completed interventional clinical trial conducted by the Centre Georges Francois Leclerc examined the expression of the HSP70 protein by EVs isolated from blood and urine of cancer patients for the early diagnosis of malignant solid tumors [75]. Indeed, recent controversial studies have shown that HSP70 was expressed only by EVs released by cancer cells, playing an important role in intercellular communication and tumor progression [77].

As evidenced by the few examples here summarized, the heterogeneity of the biofluids from which EVs can be isolated is mirrored by the heterogeneity of their potential clinical application.

### 2.2. Exosomes as Biomarkers in Clinical Applications

Most of the clinical trials falling into the “exosomes” category concern the use of vesicles in the diagnosis and prognosis (liquid biopsy) of several cancer types. However, in the majority of the registered studies, exosomes are examined as “secondary” and not disease-specific markers.

Exosome Diagnostics, Inc sponsored an observational clinical trial to study the efficacy of the Diagnostic Test “ExoDx Prostate Intelliscore (EPI)”. ExoDx Prostate is a non-invasive validated test that can be used in accordance to the ordering physician’s clinical judgments to evaluate whether a prostate needle biopsy is necessary [79]. The 532 participants enrolled in the study had a clinical suspicion for prostate cancer based in part on high prostate-specific antigen level (limit range: 2.0–10 ng/mL). The enrolled population has been divided into two cohorts: the first cohort (cohort 1), consisting of men already scheduled for an initial prostate biopsy, and the second cohort (cohort 2) composed of men without a scheduled prostate biopsy. The aims of the study were: (i) to evaluate the performance of the urine test in men already belonging to cohort 1, and (ii) to assess how the results of the urine test could influence the decision process for determining whether to perform biopsy [79].

Another application of exosomes in cancer diagnosis/characterization was investigated in an interventional, phase 2 clinical study completed by the University Medical Center of Konkuk. In this study, DNA extracted from exosomes of bronchoalveolar lavage fluid was evaluated to confirm the presence of the T790M gatekeeper mutation of the epidermal growth factor receptor (*EGFR*) in non-small cell lung cancer (NSCLC) patients [80].

Apart from the genetic material, also the exosome-associated proteins have been examined as potential disease-related biomarkers, as indicated in an observational clinical study sponsored by the University of Alabama at Birmingham dealing with Parkinson’s disease (PD). The study included 601 participants (PD patients and healthy controls) with the “Primary outcome” of finding, in exosome-derived proteomes (urine and blood), valid biomarkers associated with PD susceptibility and/or progression. The same study analyzed also whether the treatment with the multi-kinase inhibitor Sunitinib correlated with a decrease in the vesicular expression/phosphorylation of *LRRK2* [81], considering exosomes not only valuable biomarkers for disease diagnosis and prognosis, but also an important assay to confirm the effectiveness of the therapy.

Although circulating exosomes are mainly considered as valuable tools for tumor detection and progression, we will also mention few examples in which they were analyzed in other (patho)physiologic conditions.

The potential role of exosomes in blood coagulation has been examined by means of an in vitro interventional clinical study conducted analyzing the blood of 25 healthy volunteers managed by the Johann Wolfgang Goethe University Hospital [82]. Exosomes derived from red blood cell units were mixed with the blood of healthy participants to evaluate the effects on coagulation and platelet function by thromboelastometry (clotting time, clot formation, clot stability, clot lysis) and flow cytometry [82].

Sepsis represents a pathologic phenomenon in which exosomes could have a central role. Indeed, a research of Taipei Tzu Chi Hospital, Buddhist Tzu Chi Medical Foundation was the first study using blood- and urine-derived exosomes from septic patients with multiple organ failures [84]. It has been found in many animal sepsis models that a systemic infection induces multiple organ failure and it has been supposed that autophagy and apoptosis could be the cause of this event. Consequently, the observational study analyzed the expression of some biomarkers of autophagy (LC3II, mTOR, HSP70, Sequestosome 1) in exosomes collected from the serum of patients with sepsis, septic shock, or multiple organ failure. The same Hospital achieved another and similar clinical study dealing with exosomes isolated from urine and blood of septic patients and from conditioned media of human organ cell models co-cultured with lipopolysaccharide (LPS)-stimulated macrophages [68].

The involvement of exosomes following renal transplantation has been investigated in another observational clinical trial sponsored by the University Hospital of Bordeaux. The goal of the study was to verify the expression and phosphorylation of the sodium chloride co-transporter (NCC) in urinary exosomes derived from patients undergoing renal transplantation and treated with calcineurin inhibitors (CNI). The latter could be, in fact, the cause of the so-called “Gordon like” syndrome, a rare genetic disorder where NCC is over-activated and cause hypertension with other renal manifestations [85]. Two cohorts were enrolled: kidney transplanted patients treated with CNI and control healthy subjects [85].

In another completed clinical study sponsored by the Basque Country University, the expression of miRNAs in circulating exosomes isolated from the blood of 84 children with Type 2 Diabetes (T2D) risk was examined to evaluate the effect of a multidisciplinary intervention program (exercise, lifestyle and psycho-educational) on pre-adolescents with high risk to develop T2D [69].

### 2.3. Extracellular Vesicles as Therapeutic Tools in Clinical Applications

In recent years it has been demonstrated that EVs isolated from the secretome (conditioned medium) of stem/progenitor cells possess innate therapeutic properties as regenerative, immunomodulatory and anti-inflammatory tools [72]. Moreover, they are considered in the drug delivery research area for the possibility to be loaded with a therapeutic cargo and edited for targeted therapy [72].

The following paragraphs will summarize the only two completed clinical studies based on the use of natural nanoparticles in therapy.

The main aim of the clinical trial sponsored by the Medical University of Warsaw was to evaluate whether the inhibition of platelet-derived EVs could play beneficial effects in the context of acute myocardial infarction [74]. The interventional trial was based on previous observations indicating that the receptor P2Y12 is essential for platelet activation and that activated platelets release pro-inflammatory and pro-coagulant EVs [76]. The effectiveness of two P2Y12-antagonists (Ticagrelor and Clopidogrel) was evaluated in the trial, assuming that the reduced mortality observed in Ticagrelor-treated patients could be explained by greater inhibition of the release of platelet vesicles, compared to Clopidogrel. The primary outcome was to quantify the concentration of platelet EVs/mL by flow cytometry six months after the beginning of antiplatelet therapy, and the secondary outcome was the assessment of EVs exposing fibrinogen and phosphatidylserine. The main results associated to the study are reported in the “Study Results” section. Twenty-seven patients treated with Ticagrelor presented a median of 2,690,000 platelet EVs/mL, while the median number of platelet EVs/mL in 28 patients treated with Clopidogrel was higher (4,310,000), confirming that the antiplatelet drug Ticagrelor is particularly efficient in inhibiting the release of platelet EVs. In the Results section, the adverse events have also been reported. One major bleeding event from the gynecologic tract and one major bleeding event from a diabetic foot ulcer have been reported for patients treated with Ticagrelor and Clopidogrel, respectively [74].

The University Medical Centre of Ljubljana completed a clinical trial in which the autologous platelet- and extracellular vesicle-rich plasma (PVRP) was used to treat chronic inflammation of temporal bone cavities occurring after the removal of the posterior external ear canal wall by an open-technique cholesteatoma surgery. PVRP was administered to chronically inflamed radical cavities via PVRP-soaked ear wicks and the control group has been treated with standard conservative measures. The primary outcome was to evaluate the efficacy of PVRP (and so, indirectly, of EVs) in reducing the inflammation surface area [86]. In the “Results section of the study, the efficacy of PVRP treatment has been evaluated and compared to the standard control treatment based on the presence of bacteria and fungi in the temporal bone cavity. No statistically significant differences among the two cohorts of patients enrolled in the study have been reported.

### 2.4. Exosomes as Therapeutic Tools in Clinical Applications

Due to our current situation of global COVID-19 pandemic, the application of natural nanoparticles in the therapy of the SARS-CoV-2 infection has achieved big interest and some studies were focused on the application of exosomes for the treatment of this disease. An interventional phase 1 clinical study has been sponsored by the Hospital of Ruijin to evaluate the safety and efficacy of MSC-derived exosomes in severe patients affected by novel coronavirus-associated pneumonia [87]. In this context, several preclinical studies have demonstrated that mesenchymal stromal cells (MSCs) or their exosomes (MSCs-Exo) were able to reduce lung inflammation and pathological impairment resulting from different types of lung injury. The investigators considered safer to deliver MSCs-Exo rather than MSCs because intravenously administrated cells could aggregate [87]. A cohort of patients received repeated aerosol inhalations of MSCs-Exo (2.0 × 10⁸ nano vesicles/3 mL at Day 1–Day 5) and as primary outcomes they evaluated adverse reactions, severe adverse reactions and the time to clinical improvement [87]. The results associated to the study have been submitted, but are not yet publicly available, as indicated in the Results section.

An interventional study was developed to explore the safety and efficacy of aerosol inhalation of MSC-derived exosomes for the treatment of severe patients hospitalized with novel coronavirus pneumonia (NCP) [78]. The study enrolled 30 participants divided into 3 groups: two of them received standard therapy together with inhalation of 3 mL solution containing 0.5–2 × 10^10^ exosomes; the placebo group received standard therapy and twice a day inhalation of 3 mL solution free of nanoparticles. The primary outcome was to examine the presence of adverse events during the inhalation procedure and during the trial [78]. All the patients enrolled in the study did not present adverse events during the whole trial and the inhalation procedure. Moreover, in the Results section, it has been reported, among others, that at the end of the treatments, the patients treated with exosomes presented a lower level of C reactive protein compared to the placebo group, suggesting that the inflammatory processes were reduced upon exosome treatment.

Another example of therapy with MSC-conditioned media (MSC-CM) rather than isolated exosomes is the interventional clinical study sponsored by Sukma Skin Treatment [88]. This study is based on a previous pilot study in which an animal model was used to evaluate the skin ulcer healing potential of the MSC-CM. The investigators examined the therapeutic effect of Wharton’s Jelly derived MSC-CM for the treatment of patients with chronic skin ulcer [89,90], and wound healing was estimated, after two weeks, evaluating the presence of granulation tissue, size of the ulcer, edema, and erythema decrease. According to the outcomes, the researchers involved in the study stated that the natural nanoparticles present in CM could be able to trigger repair and to mediate the organogenesis of tissue-engineered organs ex vivo [88].

Among the multitude of exosome-based trials, the last example we want to report concerns the use of exosomes in vaccines for cancer immunotherapy. An interventional phase 2 “Trial of a Vaccination With Tumor Antigen-loaded Dendritic Cell-derived Exosomes” has been sponsored by the Gustave Roussy Cancer Campus of Paris [91]. The proposed immunotherapy protocol for non-small cell lung cancer (NSCLC) patients involved the metronomic dosage of Cyclophosphamide (mCTX) for 3 weeks before an immunotherapy treatment constituted by intradermal injections of tumor antigen-loaded dendritic cell-derived exosomes (Dex). Enrolled patients had an advanced unresectable NSCLC and were responding or stabilized after induction chemotherapy. The specific immunotherapy treatment was composed by two phases: the first induction-phase with injections of Dex once a week for four consecutive weeks and the second continuation-phase with injections of Dex every two weeks for six weeks. According to the investigators involved in the study, mCTX should restores T and NK cell effector functions inhibiting regulatory T lymphocytes (Treg), while Dex should be able to activate both innate and adaptive immunity. The final aim of the study was to evaluate if the therapy could improve the participant progression-free survival [91].

## 3. Conclusions

The review summarizes the main pre-clinical and clinical applications of extracellular vesicles and exosomes as biomarkers and therapeutic tools. Some technical and functional pitfalls have still to be addressed, at both preclinical and clinical levels. A more detailed molecular profiling of the vesicle cargo as well as a more stringent EV characterization should be done to further define a minimum content for specific applications. Unfortunately, most of the selected clinical trials involving the use of EVs or exosomes do not report the study results. This contributes toward complicating the appreciation of their potentialities as biomarkers or therapeutics. Nevertheless, the great interest and amount of work focused on the development of EV-based formulations bode well for their widespread application in the clinic.

## Figures and Tables

**Table 1:** Summary of clinical trials with “Extracellular Vesicles” as biomarkers or therapeutic tools.

Extracellular Vesicles					
Biomarkers			Therapeutic Tools		
Ref	Source	Application	Ref	Source	Application
[65]	Bronchoalveolar lavage fluid	Effect of cigarette smoking on EV miRNA profiles	[68]	Blood	Acute myocardial infarction
[66]	Serum	Tumor-associated hypoxia (prognostic value)	[69]	Autologous platelet- and EV-rich plasma (PVRP)	Chronic inflammation of temporal bone cavities
[67]	Cerebrospinal fluid	Specific PD-associated mutations in *LRRK2*			
[70]	Blood and Urine	Expression of the HSP70 protein in cancer patients			

**Table 2:** Summary of clinical trials with “Exosomes” as biomarkers or therapeutic tools.

Exosomes					
Biomarkers			Therapeutic Tools		
Ref	Source	Application	Ref	Source	Application
[71]	Urine	Prostate cancer	[72]	Mesenchymal Stromal Cells	SARS-Cov-2 infection
[73]	Bronchoalveolar lavage fluid	Non small Cell Lung Cancer	[74]	Mesenchymal Stromal Cells	SARS-Cov-2 infection
[75]	Blood and Urine	PD-susceptibility, progression and therapy effectiveness	[76]	Wharton’s Jelly-derived Mesenchymal Stromal Cells	Chronic skin ulcer healing
[77]	Blood	In vitro effects on blood coagulation and platelet function	[78]	Dendritic cells	Immunotherapy in Non Small Cell Lung Cancer
[79,80]	Blood and Urine	Sepsis, septic shock or multiple organ failure			
[81]	Urine	Kidney transplanted patients with calcineurin inhibitors			
[82]	Blood	Pre-adolescents with high risk to develop type 2 diabetes			

## Data Availability

Not applicable.
